# The cholesterol 24-hydroxylase activates autophagy and decreases mutant huntingtin build-up in a neuroblastoma culture model of Huntington’s disease

**DOI:** 10.1186/s13104-020-05053-x

**Published:** 2020-04-10

**Authors:** Clévio Nóbrega, André Conceição, Rafael G. Costa, Rebekah Koppenol, Raquel L. Sequeira, Ricardo Nunes, Sara Carmo-Silva, Adriana Marcelo, Carlos A. Matos, Sandrine Betuing, Jocelyne Caboche, Nathalie Cartier, Sandro Alves

**Affiliations:** 1 Department of Biomedical Sciences and Medicine, Universidade do Algarve, Faro, Portugal; 2 Centre for Biomedical Research, Universidade do Algarve, Faro, Portugal; 3 Algarve Biomedical Center, Universidade do Algarve, Faro, Portugal; 4 Center for Neuroscience and Cell Biology, University of Coimbra, Coimbra, Portugal; 5 Neuronal Signaling and Gene Regulation, Neurosciences Paris Seine, Institut de Biologie Paris Seine, Sorbonne Université, Faculté des Sciences et Ingénerie, INSERM/UMR-S 1130, CNRS/UMR 8246, 75005 Paris, France; 6 INSERM U1127, Institut du Cerveau et de la Moelle épinière (ICM), Hôpital Pitié-Salpêtrière, 47 bd de l’Hôpital, 75013 Paris, France; 7 Brainvectis, Institut du Cerveau et de la Moelle épinière (ICM), Hôpital Pitié-Salpêtrière, 47 boulevard de l’Hôpital Paris, 75646 Paris Cedex 13, France

**Keywords:** CYP46A1, Cholesterol, Neuroblastoma cells, Huntingtin, Autophagy

## Abstract

**Objective:**

Compromised brain cholesterol turnover and altered regulation of brain cholesterol metabolism have been allied with some neurodegenerative diseases, including Huntington’s disease (HD). Following our previous studies in HD, in this study we aim to investigate in vitro in a neuroblastoma cellular model of HD, the effect of CYP46A1 overexpression, an essential enzyme in cholesterol metabolism, on huntingtin aggregation and levels.

**Results:**

We found that CYP46A1 reduces the quantity and size of mutant huntingtin aggregates in cells, as well as the levels of mutant huntingtin protein. Additionally, our results suggest that the observed beneficial effects of CYP46A1 in HD cells are linked to the activation of autophagy. Taken together, our results further demonstrate that CYP46A1 is a pertinent target to counteract HD progression.

## Introduction

Huntington’s disease (HD), is an autosomal dominant inherited neurodegenerative disorder [1, 2], resulting from an abnormal CAG-repeat expansion in the coding region of the *HTT* gene, resulting in an expanded polyglutamine (polyQ) tract of the huntingtin protein (HTT) [3]. HTT protein gains a toxic function, and abnormally accumulates in the cells leading to their death [4, 5]. Clinically, HD is associated with degeneration of the striatum and cerebral cortex [6–8], and symptoms such as uncontrolled movements, chorea, dystonia and oculomotor impairments [9]. Until now there is no therapy available to delay or stop HD progression [10].

Several studies have demonstrated that cholesterol metabolism impairment in the brain could be implicated in different neurodegenerative diseases [11, 12]. Brain cholesterol is crucial for brain homeostasis and physiology, throughout development and in the course of neuronal lifespan [13, 14]. Brain cholesterol is nearly absolutely synthesized in situ [15], given that the blood-brain-barrier (BBB) precludes its efflux/influx [16]. 24-hydroxylase (CYP46A1), a key enzyme in cholesterol turn over and clearance is mostly expressed in neurons and hydroxylates cholesterol into 24S-hydroxycholesterol (24S-OHC) that freely crosses the BBB reaching peripheral circulation [17]. CYP46A1 also play an important role in neural homeostasis [17–20]. It has been shown that CYP46A1 deficiency leads to neuronal dysfunction, neuronal death, motor and cognitive impairments and that CYP46A1 overexpression improved neurodegenerative HD-related traits [21–24]. Previously, we and others showed that cholesterol metabolism is impaired in HD, and strategies to reactivate it, such as CYP46A1 expression restoration, show beneficial effects in different HD model mice, including an improvement in neuronal function, a prevention in neuronal degeneration and the recovery of motor deficits [22, 24–29]. Here, we investigated the impact of CYP46A1 overexpression in huntingtin aggregates and soluble mutant HTT (HTT-MUT) levels and characterized a potential molecular mechanism.

## Main text

### Materials and methods

#### Neuroblastoma cells culture and transfections

Mouse neuroblastoma cell line (N2a cells; ATCC^®^ CCL-131) were maintained at 37 °C, 5% CO_2_, in Dulbecco’s modified Eagle’s medium (DMEM) supplemented with 10% foetal bovine serum, 100 U/ml penicillin and 100 mg/ml streptomycin (Gibco). The different plasmids used were transfected in the cells using PEI (Polyethylenimine, #274651, Polysciences Inc.) in total concentration of 500 ng for each DNA. Cells were lysed for Western blot processing, harvested for flow cytometry analysis or fixed for fluorescence microscopy analysis, 48 h post-transfection.

#### Plasmids

Plasmids used: pEGFP-HTTQ74 (Addgene #40262, exon 1 of HTT with 319 bp) [30], pAAV-HA-CYP46A1 (provided by Brainvectis), ptfLC3-RFP-GFP (Addgene #21074) [31]. The LacZ gene was cloned in our laboratory under the control of a PGK promoter [32].

#### Western blot

The Western blot processing was performed as described previously [33]. Antibodies used for immunoblotting: mouse anti-GFP (1:1000, Biolegends, #668205), rabbit anti-LC3B (1:1000, Novus, #NB100-2220), rabbit anti-p62/SQSTM1 (1:1000, Cell Signaling, #5114), mouse anti-ubiquitin (1:1000, Cell Signaling, #3936), mouse anti-β-tubulin, (1:10,000, Sigma-Aldrich, #T7816) and mouse anti-actin (1:10,000, Sigma-Aldrich, #A5316).

#### Immunocytochemistry

Immunocytochemistry was performed based on protocols described previously [33]. Primary antibodies were incubated overnight at 4 °C (haemagglutinin (HA) tag, 1:1000, Abcam, #ab9110). Secondary antibodies were incubated for 2 h at room temperature (Alexa 594 or Alexa 647, Thermo Fisher).

#### Fluorescence microscopy analysis

Mutant HTT aggregates were directly counted in a blind fashion way in 100 random cells with 40X objective in a Zeiss Axio Imager Z2. For the aggregate’s areas assessment, the CellProfiler software was used [34]. Images were acquired with 40X objective in a Zeiss Axio Imager Z2. For the immunocytochemistry experiments, the LC3-GFP-RFP puncta was blindly counted in 100 random cells with 40X objective in a Zeiss Axio Imager Z2.

#### Chloroquine treatment

We performed the chloroquine (ChQ) treatment (SIGMA, 100 μm), an inhibitor of autolysosomal degradation, as previously described [33].

#### Ubiquitin-proteasome system (UPS) inhibition in neuroblastoma cells

Six hours prior to collection (and 42 h post-transfection), transfected N2a cells were treated with MG-132 (Sigma-Aldrich, 5 μM), an UPS inhibitor. After this treatment N2a cells were collected for western blot analysis.

#### Flow cytometry

Transfected cells were twice washed with PBS and then carefully harvested in PBS on ice. The different conditions were then processed in a flow cytometer (Becton–Dickinson FACSCalibur) using the adequate lasers, as previously described [35]. Briefly, for each condition 50,000 cells were acquired in the selected gate, and positive cells were plotted as GFP fluorescence intensity (FL1 channel, 530 ± 30 nm).

#### Statistical analysis

Statistical analyses were performed using One-way ANOVA analysis with Bonferroni’s multiple comparisons test or unpaired t-Student test. Results are expressed as mean ± SEM. Significant thresholds were set at *P < 0.05, **P < 0.001, ***P < 0.0001, ****P < *0.00001*. All analyses were performed using GraphPad Prism (GraphPad Software v6, La Jolla, USA).

### Results

**Fig. 1:**
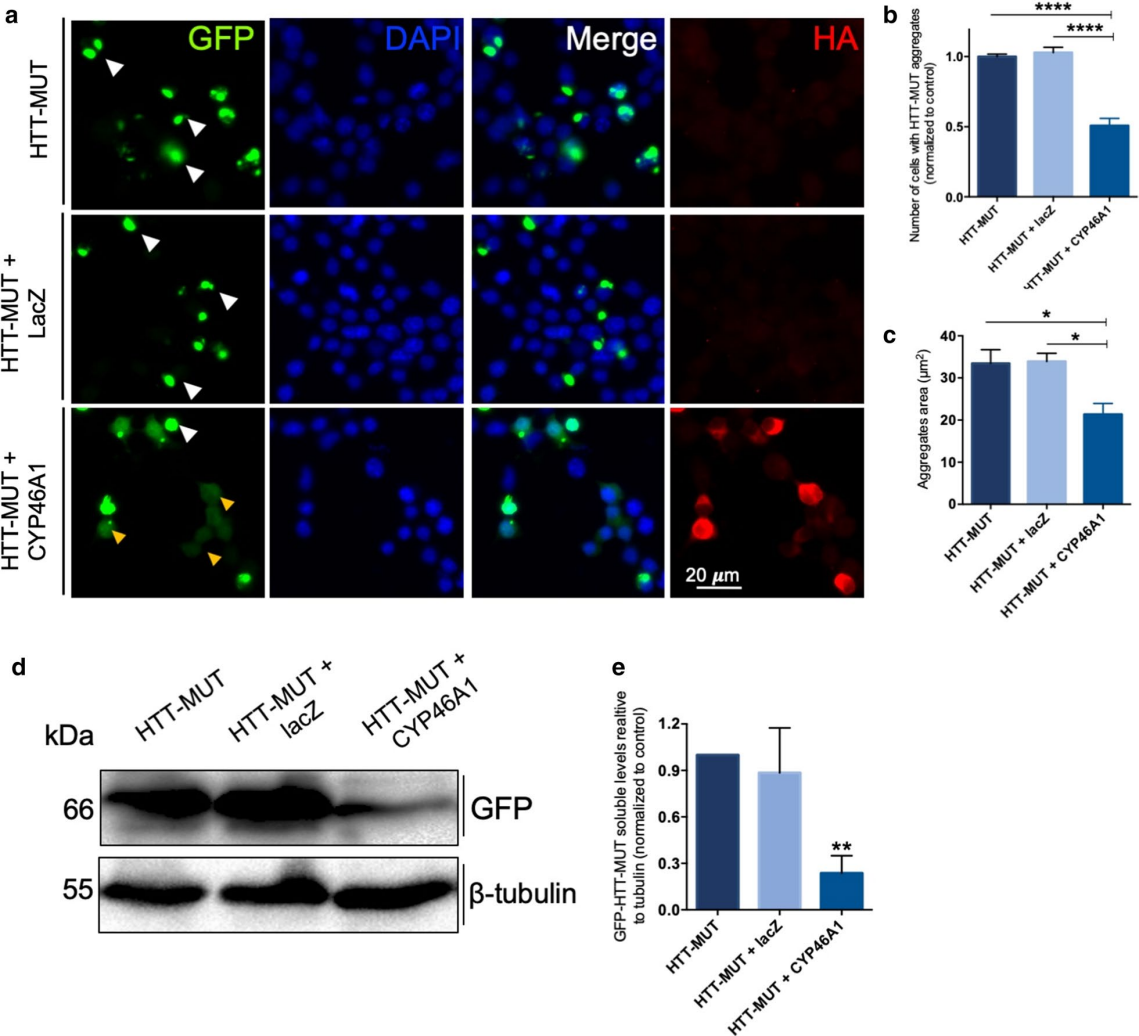
CYP46A1 overexpression decreases the number of aggregates and the levels of soluble mutant HTT. **a** N2a cells were transfected with pEGFP-HttQ74, co-transfected with pEGFP-HttQ74 and pAAV-HA-CYP46A1, and co-transfected with pEGFP-HttQ74 and LacZ, highlighting the fusion protein with GFP, the nuclei labeled with DAPI and the expression of CYP46A1 by the labeling with HA tag. For each condition, 100 cells were randomly counted. **b** The number of cells with aggregates (white arrows) upon CYP46A1 overexpression was significantly reduced compared to control conditions (note the yellow arrows with expression of HTT-MUT without aggregates). **c** The size of HTT-MUT aggregates was smaller upon CYP46A1 overexpression relatively to both control conditions. **d** Western blot analysis probed with mouse anti-GFP depicting the soluble HTT-MUT (66 kDa) and tubulin as loading control. Densitometric analysis showed that CYP46A1 expression leads to a significant reduction of the soluble HTT-MUT (**e**). Values are expressed as mean ± SEM. (**a**–**c**, *n *= *5* independent experiments; **d–e**, *n *= *4* independent experiments; One-way ANOVA with Bonferroni’s multiple comparisons test, *P < 0.05; **P < 0.001; ****P < *0.00001*)

We analyzed by immunocytochemistry the impact of CYP46A1 overexpression in neuroblastoma (N2a) cells overexpressing the exon 1 of HTT-MUT carrying 74 glutamine (Q74) fused with GFP. N2a cells were co-transfected with plasmids encoding HTT-MUT and either the plasmid encoding CYP46A1 or as control the plasmid for lacZ (Fig. 1a). Approximately 90% of all transfected cells express both plasmids, without difference in cell viability. We observed that the overexpression of CYP46A1 (labeled with HA tag) significantly reduced the number of cells with HTT-MUT aggregates compared to HTT-MUT condition and to HTT-MUT and lacZ (Fig. 1b). The aggregates area (µm^2^) was also significantly reduced upon CYP46A1 expression (Fig. 1c), as compared to HTT-MUT, and to HTT-MUT + lacZ. Next, we evaluated the impact of CYP46A1 overexpression in the protein levels of HTT-MUT. Western blot analysis of N2a lysates using anti-GFP antibody showed a statistically significant reduction in the levels of soluble HTT-MUT in the condition with CYP46A1, as compared to both control conditions (Fig. 1d–e). Altogether, these results show a robust effect of CYP46A1 in reducing the levels of HTT-MUT aggregates and protein.

**Fig. 2:**
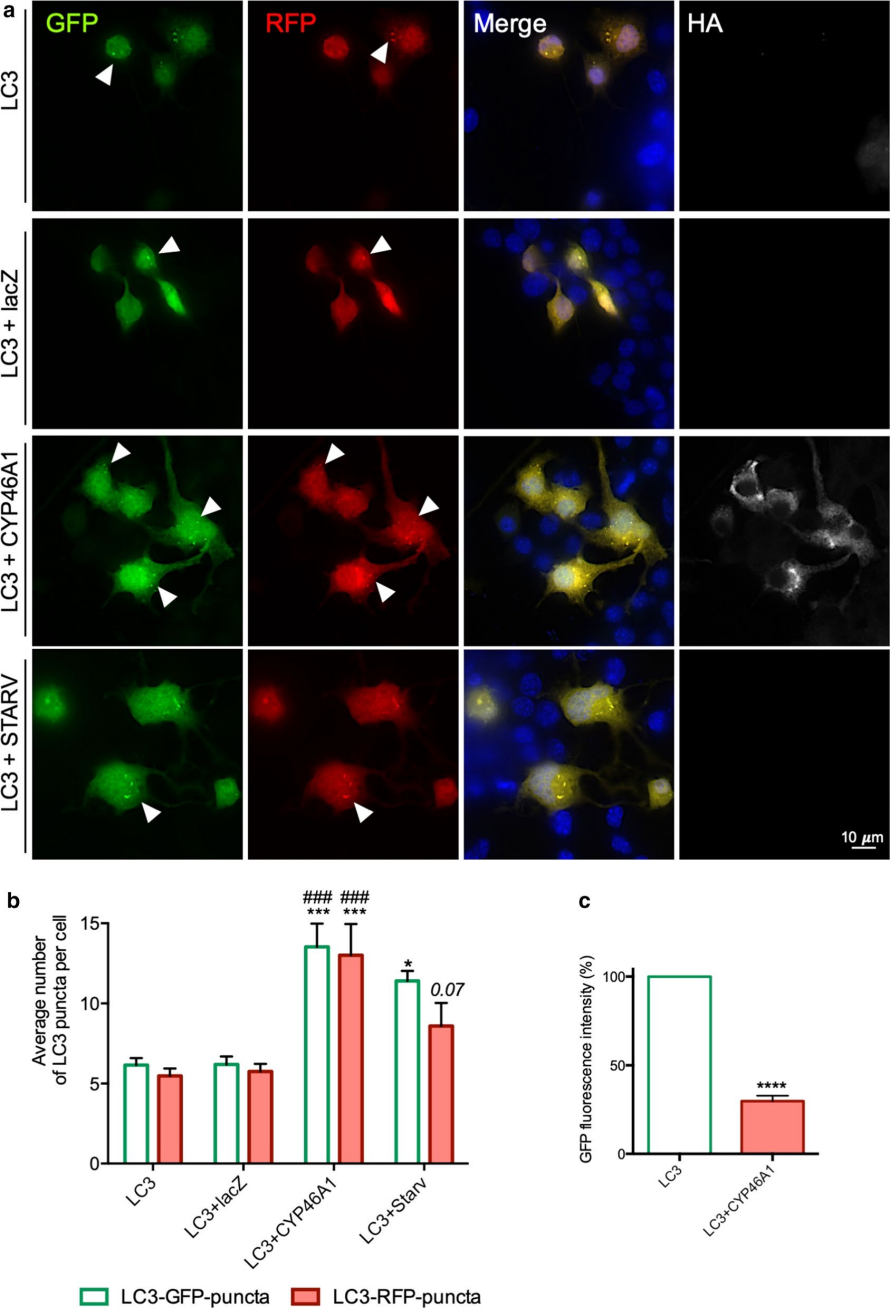
CYP46A1 overexpression significantly increases the number of LC3 puncta upon autophagy inhibition. **a** N2a cells were transfected with ptfLC3-RFP-GFP, co-transfected with ptfLC3-RFP-GFP and CYP46A1, and co-transfected with ptfLC3-RFP-GFP and lacZ. An additional condition was used, promoting starvation, as a positive control for autophagy activation. The visualization of LC3-puncta is possible upon autophagy inhibition with chloroquine in all the experimental conditions. The presence of LC3-RFP-GFP puncta (co-localized) refers to the presence of mature autophagosomes, whereas LC3-RFP puncta (without GFP) refers to autolysosomes. Representative microscopy images. **b** For each condition 100 cells were randomly counted in different microscopy fields. The number LC3-puncta per cell upon CYP46A1 overexpression was significantly increased compared to both control conditions. **c** The total fluorescence intensity (GFP) was significantly reduced upon CYP46A1 overexpression, compared to control conditions, thus suggesting an increase in the autophagic clearance. Values expressed as mean ± SEM. (**a**, **b**, *n *= *5* independent experiments, two-way ANOVA with Bonferroni’s multiple comparisons test, *P < 0.05; ***P < *0.0001;*^*###*^*P *< *0.0001* comparing to LC3 + lacZ; **c**, *n *= *3* independent experiments Unpaired Student’s *t* test, ****P < *0.00001*)

To further investigate the mechanisms of CYP46A1-mediated effects in the clearance of HTT-MUT via autophagic pathway experiments were preformed using a plasmid expressing the autophagic protein LC3B fused with RFP or GFP, and using an autophagy inhibitor (chloroquine—ChQ; Fig. 2a) [31]. We found that CYP46A1 significantly increased both LC3-GFP and LC3-RFP puncta, as compared to control conditions (Fig. 2b;). This increase was even more pronounced than the one observed in the starvation condition, which is a positive control for autophagy activation, thus suggesting that CYP46A1 is activating autophagy. We also performed a cytometry analysis using cells transfected with the LC3-GFP-RFP plasmid. We observed that CYP46A1 overexpression leads to a significant reduction in GFP fluorescence intensity compared to the control condition (Fig. 2c). As this experiment was performed in the absence of ChQ, the results suggest that CYP46A1 is activating autophagy, and promoting autophagy degradation, which could explain the observed reduction in the levels of HTT-MUT aggregates and protein.

**Fig. 3:**
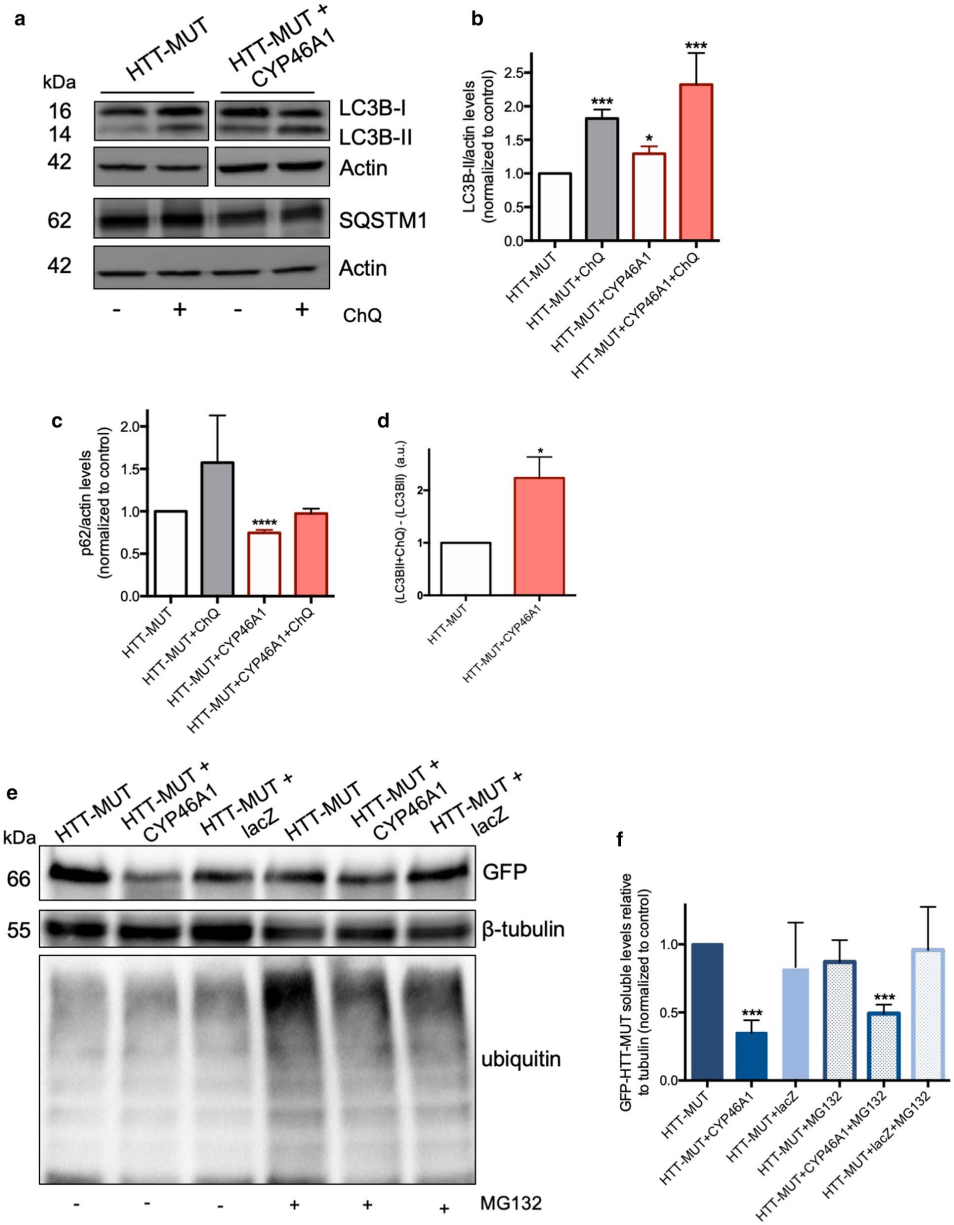
CYP46A1 overexpression promotes the activation of autophagy, independently of the UPS system. **a** N2a cells were transfected with pEGFP-HTTQ74, co-transfected with pEGFP-HTTQ74 and CYP46A1. Western blots were probed with rabbit anti-LC3B, rabbit anti-p62/SQSTM1, and mouse anti-actin. **b** The densitometric analysis showed that CYP46A1 expression leads to a significant increase in the LC3B-II levels and **c** to a significant reduction in the SQSTM1/p62 levels, both compared to the experimental control. The results with chloroquine inhibition of autophagy follow the same trend, suggesting a robust autophagy activation upon CYP46A1 overexpression. **d** This activation is also supported by a significant increase in the autophagic net flux. **e** Representative western blot probed for GFP of protein lysates from N2a cells of the different experimental conditions, with and without the proteasome inhibitor MG132 (5 M). The western blot was also probed for tubulin, and poly-ubiquitin to highlight the UPS inhibition. **f** The densitometric analysis showed that CYP46A1 expression decreases GFP levels (HTT-MUT), with and without ubiquitin–proteasome system inhibition. *(n *= *4* independent experiments, Unpaired Student’s t-test; one-way ANOVA with Bonferroni’s multiple comparisons test, *P < 0.05; **P < 0.001; ***P < *0.0001*)

Next, we analyzed the impact of CYP46A1 in several autophagy markers by western blot (Fig. 3a). Overexpression of CYP46A1 significantly increases LC3B-II levels compared to control condition (Fig. 3b), thus suggesting an activation of autophagy. Moreover, in the presence of ChQ the results follow the same trend. SQSTM1/p62 is a protein involved in the degradation of autophagy via a specific interaction between p62 and LC3 [44]. Importantly, a significant reduction in the SQSTM1/p62 levels was observed upon CYP46A1 overexpression, as compared to the control condition (Fig. 3c), suggesting an increase in autophagic activity. Next, we calculated the autophagic flux by measuring the LC3B turnover assay, which is used to quantify the amount of LC3B-II that is delivered to the lysosomes and thus the autophagy dynamics. We found that CYP46A1 overexpression leads to a significant increase in the autophagic flux, as compared to the control condition (Fig. 3d). To assess selective autophagy activation by CYP46A1, we performed an experiment inhibiting the ubiquitin–proteasome system (UPS) using MG132, which leads to the accumulation of polyubiquitinated proteins (Fig. 3e). The CYP46A1 overexpression reduced the levels of HTT-MUT independently of UPS inhibition (Fig. 3f). Altogether, all these results suggest that CYP46A1 is able to activate autophagy pathway in a cellular model of HD.

### Discussion

Impaired brain cholesterol metabolism has been broadly associated with neurodegenerative disorders. We and others showed that strategies aiming at restoring cholesterol metabolism using different strategies are beneficial in AD, SCA and HD mouse models [22, 24, 36, 37]. Local synthesis/efflux of cholesterol is essential for the structural regulation of cells and membranes within the brain, being implicated in signal transduction, release of neurotransmitters and membrane trafficking [38]. Cholesterol has not the ability to cross the blood–brain-barrier (BBB). In order to be eliminated by the brain, therefore allowing brain cholesterol homeostasis, the excess of cholesterol is converted by the 24-hydroxylase (exclusively expressed in neurons) into 24-OHC that crosses the BBB, allowing brain cholesterol efflux [39, 40].

We have previously shown that CYP46A1 expression was able to reduce the number and size of intranuclear protein aggregates within the striatum of HD mouse models and improve motor impairment [22], emphasizing the neuroprotective effect conferred by CYP46A1 in HD mice [22, 24]. Here, we further show that CYP46A1 is able to decrease the number and size of HTT-MUT aggregates within a neuroblastoma cellular model of HD. Furthermore, reduction of soluble HTT-MUT was decreased upon CYP46A1 overexpression. Autophagy is impaired in HD [41] and activating autophagy by expressing CYP46A1, may in part explain how inclusions and protein levels are decreased. We previously showed in a SCA3 in vitro and in vivo models that CYP46A1 robustly increased the autophagic flux, i.e., augmented the levels of LC3-II and decreased the p62/SQSTM1 levels [36].

It is important to point that several studies have previously shown that the activation of autophagy, both using molecular or pharmacological approaches, is effective in removing HTT-MUT, and other mutated polyglutamine proteins, being therefore a privileged target for the development of effective therapeutic approaches [42–44, 33, 45–49].

Altogether, our data suggest that CYP46A1 degrades HTT-MUT via autophagy and that CYP46A1 may be a good target to alleviate HD progression. Therefore, our findings and our previous studies suggest that CYP46A1 is implicated in HTT-MUT degradation via autophagy.

## Limitations

Cellular models display several limitations and in vivo studies must be perform as robust platforms for disease modelling supporting CYP46A1 in the activation of autophagy. Furthermore, additional studies of autophagy could be performed in this cellular model, including with additional controls, to further strengthen the observed results.

## Data Availability

All the raw data generated are available upon reasonable request to corresponding author.

## Abbreviations

HD: Huntington’s disease
HTT: Huntingtin
BBB: Blood–brain-barrier
DMEM: Dulbecco’s modified Eagle’s medium
PEI: Polyethylenimine
HA: Haemagglutinin
ChQ: Chloroquine
UPS: Ubiquitin–proteasome system
N2a: Neuroblastoma cells
